# Genome-wide identification of rubber tree pathogenesis-related 10 (PR-10) proteins with biological relevance to plant defense

**DOI:** 10.1038/s41598-024-51312-3

**Published:** 2024-01-11

**Authors:** Rawit Longsaward, Unchera Viboonjun

**Affiliations:** 1 Department of Plant Pathology, Faculty of Agriculture, Kasetsart University, Bangkok, 10900 Thailand; 2 Department of Plant Science, Faculty of Science, Mahidol University, Bangkok, 10400 Thailand

**Keywords:** Computational biology and bioinformatics, Plant sciences

## Abstract

Pathogenesis-related 10 (PR-10) is a group of small intracellular proteins that is one of 17 subclasses of pathogenesis-related proteins in plants. The PR-10 proteins have been studied extensively and are well-recognized for their contribution to host defense against phytopathogens in several plant species. Interestingly, the accumulation of PR-10 proteins in the rubber tree, one of the most economically important crops worldwide, after being infected by pathogenic organisms has only recently been reported. In this study, the homologous proteins of the PR-10 family were systemically identified from the recently available rubber tree genomes in the NCBI database. The sequence compositions, structural characteristics, protein physical properties, and phylogenetic relationships of identified PR-10 proteins in rubber trees support their classification into subgroups, which mainly consist of Pru ar 1-like major allergens and major latex-like (MLP) proteins. The rubber tree PR10-encoding genes were majorly clustered on chromosome 15. The potential roles of rubber tree PR-10 proteins are discussed based on previous reports. The homologous proteins in the PR-10 family were identified in the recent genomes of rubber trees and were shown to be crucial in host responses to biotic challenges. The genome-wide identification conducted here will accelerate the future study of rubber tree PR-10 proteins. A better understanding of these defense-related proteins may contribute to alternative ways of developing rubber tree clones with desirable traits in the future.

## Introduction

Among 17 subclasses of pathogenesis-related (PR) proteins in plants, pathogenesis-related 10 (PR-10) is the only group of small intracellular PR proteins. The class 10 PR proteins have been reported to be induced in response to pathogen attack, environmental stresses, as well as growth and development^1^. According to Pfam PF00407, protein members in the PR-10 family have been identified in a wide range of plant species, from gymnosperms to angiosperms. The conserved protein folding characteristic of the PR-10 family is a “Bet v onefold”, which consists of an N-terminal short α-helix followed by paralleled 6–7 β-strands and a long C-terminal α-helix^2^. This kind of folding allows PR-10 proteins to bind to several small ligands, which are suggested to be hydrophobic, including flavonoids, fatty acids, and phytohormones^3^. A recent update on PR-10 proteins with respect to metabolite biosynthesis suggests they play an important role in the molecular pathway of host defenses^4^. Several PR-10 protein members have been proven to perform enzymatic activities, such as norcoclaurine synthase in plants from the order Ranunculales^5–8^ and ribonuclease activity in over 20 species across the plant kingdom^9^.

Although only a small proportion of PR-10 protein members have been validated for their biochemical activities^4^, the functions linked to their differential expression during stress have been extensively reported. Concerning anti-phytopathogen performance, the upregulation of PR-10 was shown to be a response in host plants against a wide range of organisms, including viruses, bacteria, fungi, oomycetes, and nematodes. The antiviral activities of PR-10 proteins have been reported in hot peppers against the tobacco mosaic (TMV-P_0_) virus^10^ and in *Nicotiana benthamiana* plants against Beet necrotic yellow vein virus^11^. A grape PR-10 protein was expressed in response to the bacterial pathogen *Pseudomonas syringae* pv. *pisi*^12^. Similarly, oomycetes-infected eucalyptus and cacao showed an increase in PR-10 proteins as a host defense response^13,14^. Moreover, PR-10 proteins have also been shown to contribute to plant immunity against nematode invasions^15,16^. In response to fungal infection, PR-10 proteins are highly induced in parsley^17^, wheat^18^, and especially rubber trees^19,20^.

The rubber tree, or *Hevea brasiliensis* (Willd. ex A.Juss.) Mull.Arg., is an important crop plant of the family Euphorbiaceae. It is the main source of natural latex, a raw material used in hundreds of industrially produced products and ubiquitous in daily life. It is primarily grown in tropical regions, especially in Southeast Asia and Africa, but these areas are now challenged not only by climate change but also by the global spread of pathogenic microorganisms^21–23^. Scientific research on rubber tree-pathogen interactions has been valuable for understanding and managing problematic diseases, i.e., white root rot disease^24^ and powdery mildew^25^. Therefore, reports on differentially expressed PR-10 proteins in rubber trees under disease conditions are noteworthy, largely because of the economic importance of this plant species, as evidenced by our recently reported novel PR-10 protein, which was highly upregulated in rubber trees that were infected with white root rot disease fungus^20^.

Recent advances in cutting-edge technology have allowed researchers to annotate members of the PR-10 protein family in several plant species through genomic and transcriptomic data^26–28^. Although several PR-10 proteins in the rubber tree are noted as key proteins in response to pathogenic attack^19,20^, we still lack a systematic identification and classification of rubber tree PR-10 proteins at the protein family level and a comprehensive discussion of their role in plant defense. In this study, we identify and report the homologous proteins of PR-10 candidates that were available in the genomes of two rubber tree cultivars, GT-1^29^ and MT/VB/25A 57/8 according to a recent NCBI BioProject PRJNA976717^30^ in the NCBI database. The sequence compositions, structural characteristics, protein physical properties, and phylogenetic relationships of identified rubber tree PR-10 proteins are discussed and used to support their classification into subgroups according to the previous assumptions of the PR-10 protein family. The gene expression and potential roles of rubber tree PR-10 proteins in plant immunity are also discussed.

## Materials and methods

### In silico identification of rubber tree PR-10 protein

The amino acid sequences of proteins in the pathogenesis-related 10 protein family (Pfam PF00407) from the rubber tree (*Hevea brasiliensis*), Arabidopsis (*Arabidopsis thaliana*), rice (*Oryza sativa*), and birch (*Betula pendula*) were downloaded from the InterPro database (https://www.ebi.ac.uk/interpro/entry/pfam/PF00407/, accessed on July 26, 2023) as query sequences for homolog searching. Protein sequences from these species could encompass the query sequences from the rubber tree itself, as well as those from other model species of dicots, monocots, and the original species of the PR-10 protein family. Then, homologs in rubber tree genomes were searched using BLASTp against the organism id: 3981 in the NCBI database (https://blast.ncbi.nlm.nih.gov/Blast.cgi?PAGE=Proteins, accessed on July 26, 2023), which yielded two main genomes from rubber tree cultivars, isolate MT/VB/25A 57/8^30^ and cultivar GT1^29^. Non-redundant hit sequences with an *E*-value < 0.01, max score > 50, and a sequence length greater than 100 amino acids were considered hit candidates from the BLASTp search. The hit candidates were then examined using the Hidden Markov Model (HMM) through hmmscan in HmmerWeb version 2.41.2^31^ (https://www.ebi.ac.uk/Tools/hmmer/search/hmmscan, accessed on July 29, 2023) with Gathering Cut-offs in order to scan for the presence of a Bet v 1-domain (PF00407) or Bet v 1-like superfamily (Superfamily 55,961). Accordingly, proteins with a Bet v 1-domain or Bet v 1-like superfamily by hmmscan were then identified as PR-10 proteins in rubber tree genomes. The protein candidates were then matched with accessions in the UniProt database to further acquire the reported protein properties and to download the AlphaFold-predicted 3D structures (https://alphafold.ebi.ac.uk, accessed on August 28, 2023).

### Structural and motif analysis

The protein characteristics reported with the NCBI accessions and matched UniProt accessions were noted, including the length of amino acids and the location of the gene on the chromosome. The molecular weight and pI of proteins were predicted using ExPASy (https://web.expasy.org/compute_pi/, accessed on August 15, 2023). Signal peptides were predicted with SignalP 6.0 (https://services.healthtech.dtu.dk/services/SignalP-6.0/, accessed on August 15, 2023). The distribution of identified PR-10 genes on rubber tree chromosomes was illustrated via MG2C version 2.1 (http://mg2c.iask.in/mg2c_v2.1/, accessed on August 29, 2023)^32^.

The motif composition of each protein sequence was then predicted by searching for novel motifs through the MEME Suite^33^ version 5.5.3 (http://meme-suite.org/tools/meme, accessed on August 26, 2023), with the parameter settings adjusted from the report of Islam and colleagues^28^ as follows: any number of repetitions (anr) site distribution; 10 motifs were predicted; the motif width was 10 to 30 amino acids; other parameters remained at default. The discovered motifs were rechecked by comparing them to the motifs in the PROSITE database (https://prosite.expasy.org/) using the Tomtom motif comparison tool^34^ (https://meme-suite.org/meme/tools/tomtom, accessed on August 26, 2023). Known motifs were manually searched in identified rubber tree PR-10 protein sequences against the PROSITE database using the ScanProsite tool (https://prosite.expasy.org/scanprosite/, accessed on August 30, 2023).

The hydrophobic cluster of each AlphaFold-predicted 3D structure was analyzed using ProteinTools^35^ (https://proteintools.uni-bayreuth.de/clusters/, accessed on September 1, 2023). The volume of each protein structure was measured through ProteinVolume v. 1.3^36^ (https://gmlab.bio.rpi.edu/PVolume.php, accessed on September 1, 2023), using a starting volume probe radius = 0.08 Å, ending volume probe radius = 0.02 Å, and surface probe minimum distance = 0.1 Å. The variation of these structural characters was analyzed by the principal component analysis (PCA) calculator (https://www.statskingdom.com/pca-calculator.html, accessed on September 3, 2023).

### Phylogenetic analysis

The protein sequences were aligned using the MUSCLE algorithm^37^ in MEGA 11 software^38^ and were used for phylogenetic tree construction using maximum likelihood (ML) in PhyML version 3.0^39^ (http://www.atgc-montpellier.fr/phyml/, accessed on August 21, 2023) with SMS automatic model selection^40^ and a bootstrap of 500 reconstructed replicates. The resulting tree was enhanced using iTOL^41^ (https://itol.embl.de/, accessed on August 24, 2023) and Microsoft PowerPoint.

## Results

### In silico identification of PR-10 proteins in rubber tree genomes

**Figure 1:**
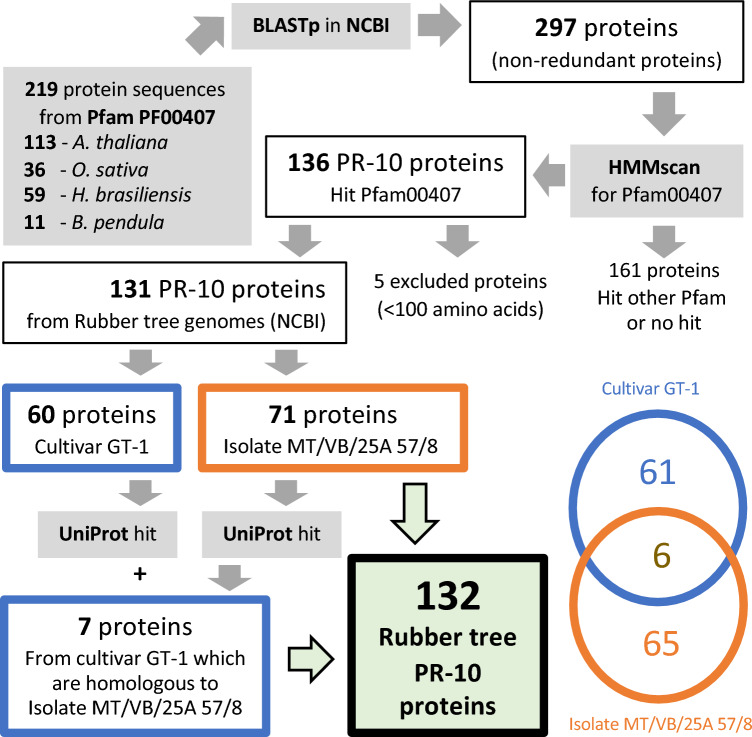
The in silico genome-wide identification process involved identifying PR-10 protein homologs in the rubber tree genomes of isolate MT/VB/25A 57/8 and cultivar GT-1 using available databases, NCBI and UniProt. A total of 132 protein candidates were identified, comprising 65 proteins from rubber tree isolate MT/VB/25A 57/8, 61 proteins from rubber tree cultivar GT-1, and 6 identical protein candidates from the two rubber tree cultivars.

A total of 219 Bet v 1-related query sequences (Fig. 1, Table S1) were selected from Pfam PF00407, including 59 sequences from *H. brasiliensis* (rubber tree), 113 sequences from *Arabidopsis thaliana*, 36 sequences from *Oryza sativa*, and 11 reviewed sequences from the original species *Betula pendula*. From BLASTp, using query sequences against the NCBI database selectively for the rubber tree organism id: 3981 (Table S2), we obtained 297 non-redundant sequences of hit candidates from two rubber tree genomes, isolate MT/VB/25A 57/8 and cultivar GT1 (Fig. 1, Table S3). Using an HMM scan, 136 proteins were confirmed to contain the Bet v 1 domain (PF00407) with significant *E*-values (Tables S4, S5). The 5 identified proteins with amino acid lengths less than 100 residues were excluded (Table S5).

Among the 131 PR-10 proteins found when BLASTing against the two study genomes, 71 proteins are from isolate MT/VB/25A 57/8 (Table S6), and 60 proteins are from cultivar GT1 (Table S7). Upon matching these 131 PR-10 proteins against the UniProt database, the proteins from cultivar GT-1 were linked to the corresponding UniProt accessions of each protein (Tables S7), whereas the proteins from isolate MT/VB/25A 57/8 were hit with the PR-10 protein from either rubber tree or cassava (*Manihot esculenta*) with different percentages of identity and coverage (Table S6). Seven other PR-10 proteins from cultivar GT-1 were then further included as they matched the candidate from isolate MT/VB/25A 57/8 in the previous step (Fig. 1). The proteins from the two rubber tree cultivars were manually checked to see if any were identical proteins found in both cultivar GT-1 and isolate MT/VB/25A 57/8. As a result, 6 of 138 proteins were identical among the two genomes.

Overall, a total of 132 non-redundant protein candidates were identified as rubber tree PR-10 proteins in this study (Fig. 1), including 65 proteins from isolate MT/VB/25A 57/8 (Table S6), 61 proteins from cultivar GT-1 (Table S7), and 6 protein candidates found in both cultivars (Table S8). Depending on the information available for each protein, different analyses could be conducted, as summarized in Table 1.

**Table 1:** An overview of the criteria employed in protein categorization, the corresponding quantities within each category, and the diverse analyses conducted for each protein category.

Criteria	Number of proteins	Analyses conducted
All identified rubber tree PR-10 proteins	132	Sequence analyses Phylogenetic analysis Motif analyses Chromosomal mapping
Identified rubber tree PR-10 proteins with associated UniProt accession (from cultivar GT-1)	67	GO terms analysis AlphaFold2-predicted 3D structure observation
Identified rubber tree PR-10 proteins with associated UniProt accession which showed a typical Bet v 1 fold as predicted by AlphaFold2	31	Protein volume analysis Hydrophobic cluster analysis

### Phylogeny among the identified rubber tree PR-10 proteins

**Figure 2:**
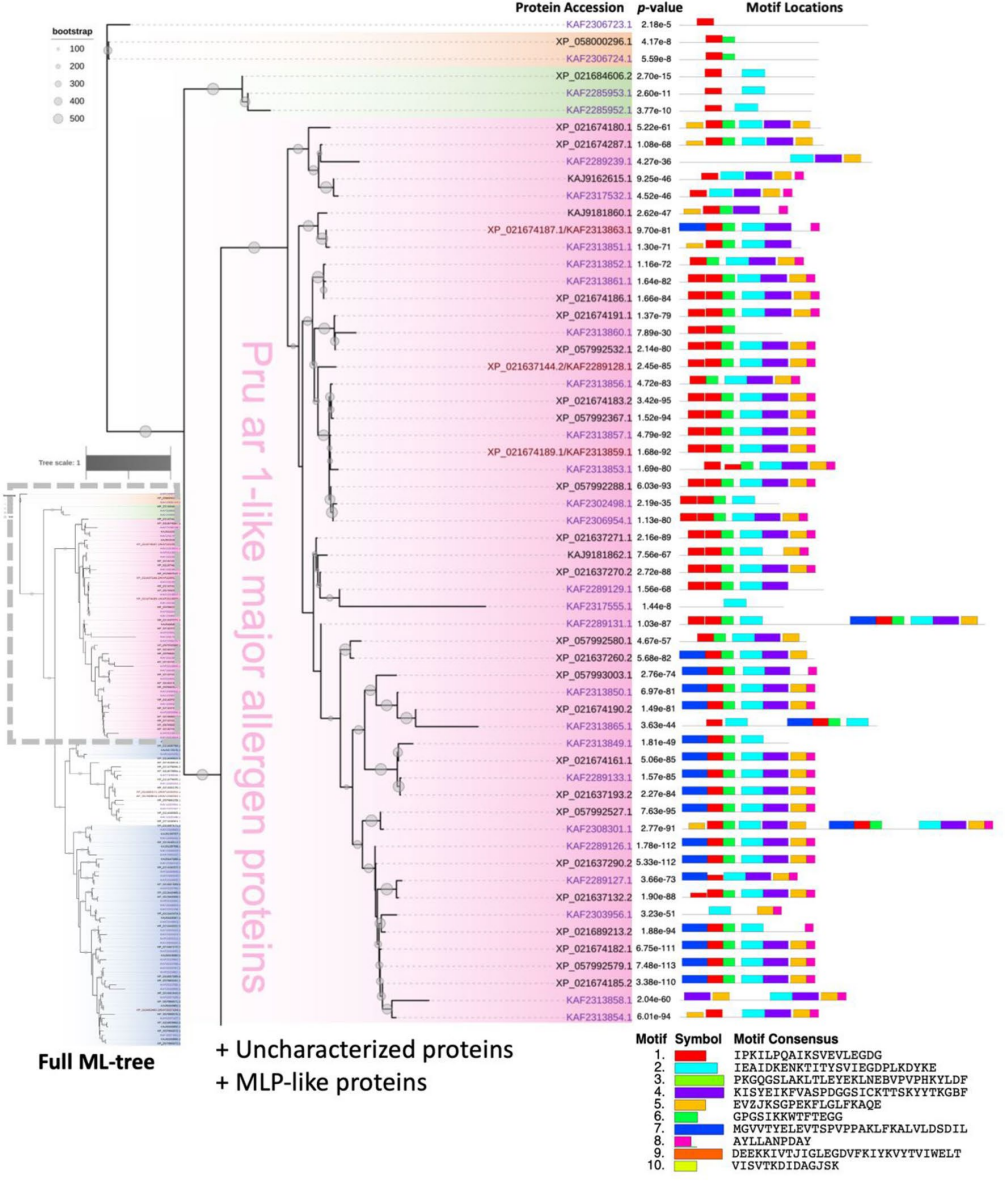
The top half of the evolutionary relationship of identified PR-10 proteins from two rubber tree cultivars, GT-1 and MT/VB/25A 57/8, inferred using maximum likelihood (ML) with 500 bootstraps (Figure S1). The protein accessions from cultivar GT-1, isolate MT/VB/25A 57/8, or both cultivars are text-coloring as purple, black, and brown, respectively. Five protein subgroups were identified: Norbelladine synthase proteins (orange), phytohormone-binding proteins (green), Pru ar 1-like allergens (pink), uncharacterized proteins, and major latex protein (MLP)-like proteins. Motif analysis using the MEME tool revealed 10 novel motifs (bottom right corner), and the *p*-values of motif predictions and the motif locations on the protein sequences are shown to the right of each identified PR-10 protein. A detailed motif analysis by the MEME tool is presented in Supplementary 1.

**Figure 3:**
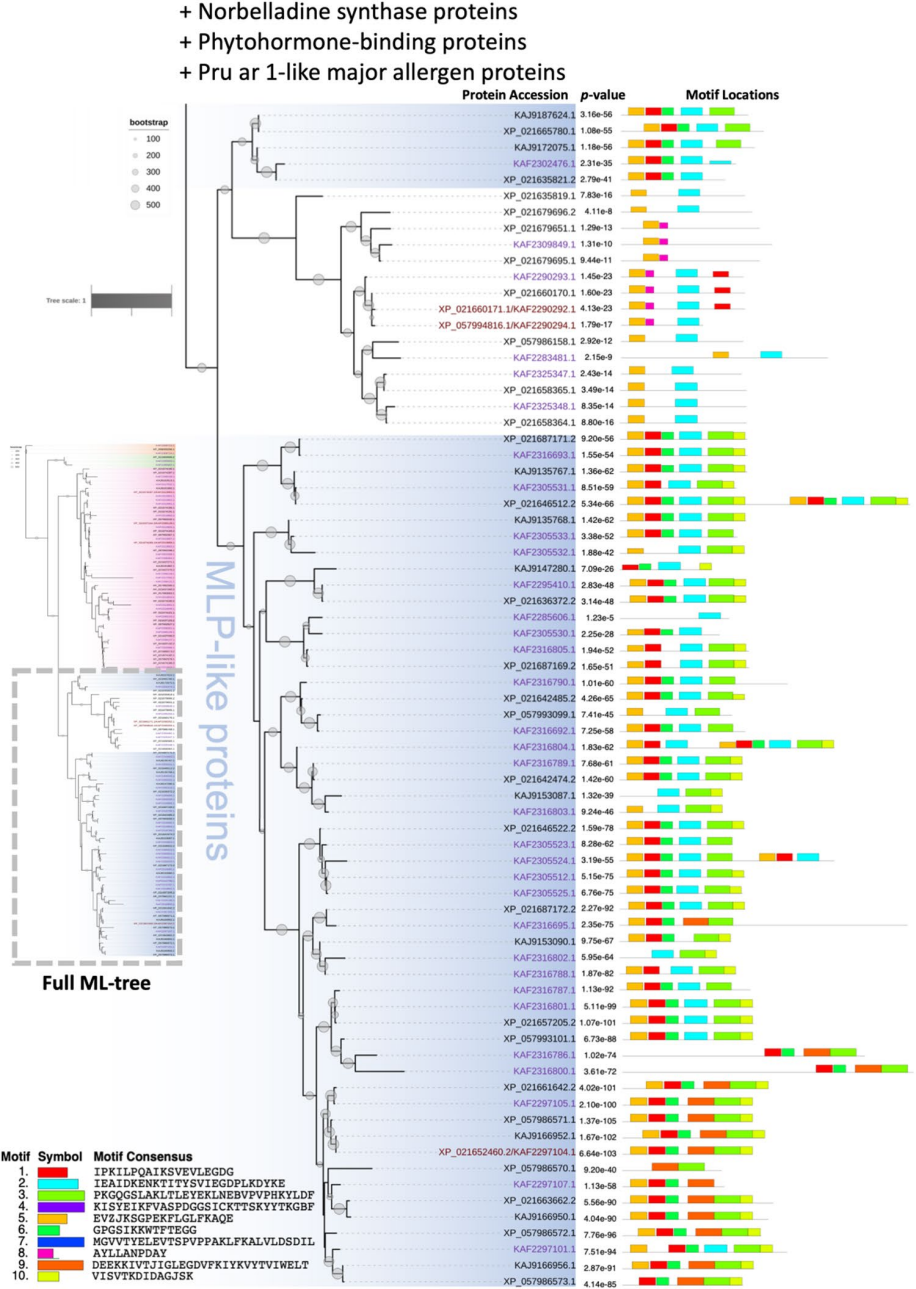
The bottom half of the evolutionary relationship of identified PR-10 proteins from two rubber tree cultivars, GT-1 and MT/VB/25A 57/8, inferred using maximum likelihood (ML) with 500 bootstraps (Figure S1). The protein accessions from cultivar GT-1, isolate MT/VB/25A 57/8, or both cultivars are text-coloring as purple, black, and brown, respectively. Five protein subgroups were identified: Norbelladine synthase proteins, phytohormone-binding proteins, Pru ar 1-like allergens, uncharacterized proteins (white), and major latex protein (MLP)-like proteins (blue). Motif analysis using the MEME tool revealed 10 novel motifs (bottom left corner), and the *p*-values of motif predictions and the motif locations on the protein sequences are shown to the right of each identified PR-10 protein. A detailed motif analysis by the MEME tool is presented in Supplementary 1.

Maximum likelihood evolutionary trees were constructed to infer the phylogenetic-based relationship between the identified PR-10 proteins from both genomes using a bootstrap of 500 replicates, and the consensus tree was obtained (Figure S1). Based on the protein name from the rubber tree isolate MT/VB/25A 57/8 (Figure S1), rubber tree PR-10 proteins were clustered into 2 major groups, one consisting of major allergen Pru ar 1-like proteins (Fig. 2) and another consisting of major latex protein (MLP)-like proteins (Fig. 3). Additionally, three minor groups (a group of three phytohormone binding proteins (PhBPs), a group of two norbelladine synthase proteins, and a group of MLP423/uncharacterized proteins) were clustered with the two major groups (Figs. 2, 3, and S1).

### The physicochemical properties and chromosomal locations of identified rubber tree PR-10 proteins

The lengths of the identified PR-10 proteins from rubber trees ranged from 100 to 373 amino acids, corresponding to molecular weights of 11.11 to 41.52 kDa (Tables S6, S7, S8). Overall, most of the proteins had a length of 150–160 amino acids, were 15–20 kDa, and were acidic with pI values below 7. None of the 132 proteins examined had signal peptides, as predicted by SignalP 6.0 (Tables S6, S7, S8).

The genome of rubber tree cultivar GT1 was developed using single-molecule real-time sequencing (SMRT) and Hi-C technologies to anchor the ∼1.47-Gb genome assembly^29^, while the recent genome from the wild isolate MT/VB/25A 57/8 used the SMRT sequencing and Illumina HiSeq technologies to yield the high-quality 1.72-Gb genome assembly^30^. The genome sequences were anchored to the 18 chromosomes of each rubber tree cultivar^29,30^.

**Figure 4:**
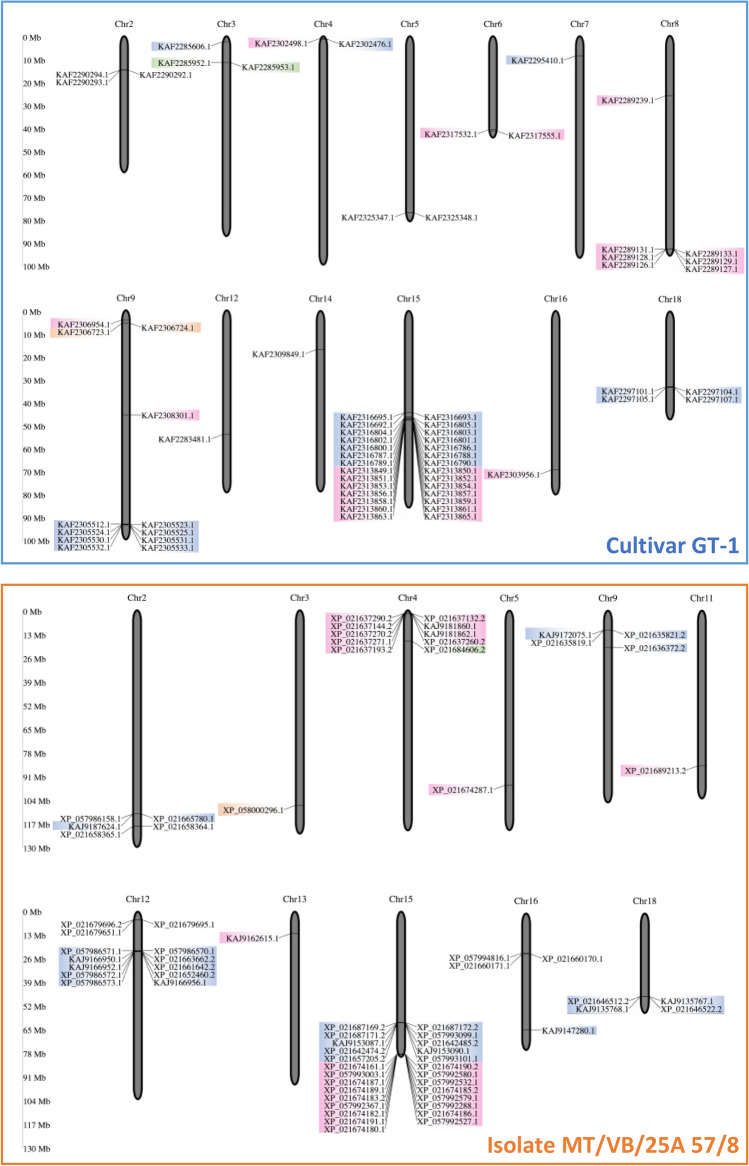
Chromosomal mapping showing the locations of the identified *PR-10* genes from rubber tree cultivar GT1 and isolate MT/VB/25A 57/8 on their respective chromosomes. The detailed genomic location of each gene is listed in Tables S6 and S7. Colors represent the different protein subgroups according to the phylogenetic tree in Figs. 2 and 3.

The locations of the genomic regions encoding rubber tree PR-10 were investigated and plotted (Fig. 4). We found that among the 67 PR10-encoding genes found on the genome of rubber tree cultivar GT1, 28 genes were clustered on chromosome 15 (Fig. 4), suggesting that many of them may have arisen from tandem duplication events^29^. The others were clustered on chromosome 9 (12 genes), chromosome 8 (7 genes), chromosome 18 (4 genes), chromosomes 2 and 3 (3 genes each), chromosomes 4, 5, and 6 (2 genes each), and chromosomes 7, 12, 14, and 16 (one gene each) (Fig. 4). Also, the PR10-encoding genes found on the genome of isolate MT/VB/25A 57/8 were majorly clustered on chromosome 15 (27 genes) (Fig. 4). The others were clustered on chromosome 12 (13 genes), chromosome 4 (10 genes), chromosome 2 (5 genes), chromosomes 9, 16, and 18 (4 genes each), and chromosomes 3, 5, 11, and 13 (one gene each) (Fig. 4).

### The motif analysis of the identified rubber tree PR-10 proteins

A total of 10 novel motifs were predicted from the amino acid sequences of all 132 proteins from two rubber tree cultivars using the MEME motif discovery server (Supplementary 1). The novel motif locations could be classified into several patterns according to their names and motif arrangement. Overall, variability in the types, locations, and frequencies of the motifs was noticed among sequences, but such variation was strongly associated with the classification of PR-10 proteins based on the ML phylogenetic tree (Figs. 2, 3, and S2). A unique motif localization pattern was found for each protein subgroup.

The Pru ar 1-like proteins had mostly conserved novel motif locations, while their N-terminal patterns were found to be either motif 7;1 or motif 1;1. Four potential homodimers of rubber tree PR-10 protein were noticed, including Pru ar 1-like accessions KAF2308301.1 and KAF2289131.1 (Fig. 2), and MLP-like accessions XP_021646512.2 and KAF2316804.1 (Fig. 3). The most conserved novel motifs found throughout the genomes were motif 2 (cyan) and motif 1 (red), respectively (Figs. 2, 3, and S2). These were the only two motifs predicted to be present in the central region of 3 phytohormone-binding proteins (Fig. 2). Motif 2 was found at up to 115 sites on 108 sequences, but was absent in several uncharacterized proteins and MLP-like proteins, as well as in norbelladine synthase proteins (Figs. 2, 3, and S2). It is clearly shown that the MLP-like proteins lacking motif 2 was substituted by motif 9 (orange) in particular locations (Figs. 3 and S2).

Motif 1 was found at up to 135 sites on 109 sequences but was absent in several proteins, including in 12 out of 15 uncharacterized proteins (Fig. 3). Although the uncharacterized proteins mostly lacked motif 1, they all exhibited motif 5 at the N-terminal, similar to most of the MLP-like proteins. In contrast, the Pru ar 1-like proteins exhibited motif 5 at the C-terminal. Motif 5 was not observed in the norbelladine synthase proteins or phytohormone-binding proteins (Figs. 2, 3, and S2).

**Figure 5:**
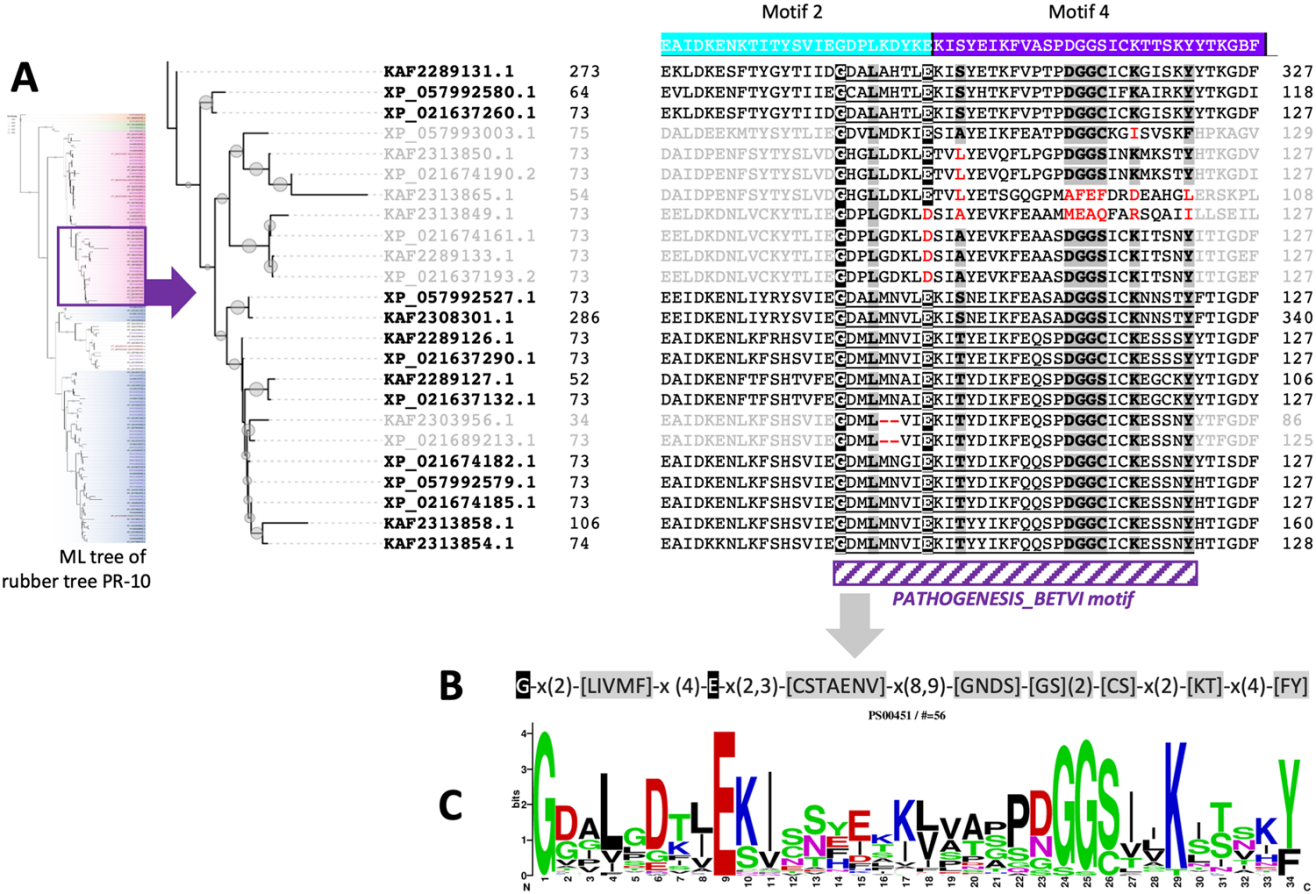
Analysis of the Bet v 1 signature motif (PATHOGENESIS_BETVI motif) on identified PR-10 proteins from rubber trees. (**A**) MUSCLE alignment of the identified rubber tree PR-10 protein sequences containing the Bet v 1 signature motif and related proteins in the subclade according to the Maximum Likelihood tree (Figure S1). The Bet v 1 signature motif is bolded and underlined, black highlight indicates the conserved residues, gray highlight indicates residues that match with one of the amino acids in the residue on the consensus sequence, red characters indicate substitutions and InDels of the key residues in the consensus sequence. (**B**) The consensus sequence of the Bet v 1 signature motif. (**C**) The sequence logo of the Bet v 1 signature motif from Prosite ID: PS00451.

The 10 discovered motifs were compared to known motifs in the PROSITE database using the Tomtom motif comparison tool, but none of the matched motifs were found to be identical. Direct searching using the PrositeScan tool is another strategy that can be used to identify known motifs present on the protein sequences of interest. Each of the 132 identified rubber tree PR-10 proteins was further submitted to the PrositeScan directly. Subsequently, 14 proteins were identically matched to the PATHOGENESIS_BETV1 motif (Prosite ID: PS00451), which is the signature motif of the pathogenesis-related 10 (PR-10) protein family (Fig. 5). These 14 proteins with the PATHOGENESIS_BETV1 motif signature were all in the Pru ar 1-like protein subgroup. In contrast, other members in this subgroup showed insertions/deletions and amino acid substitutions, resulting in imperfect matches using the PrositeScan tool (Fig. 5A).

### In silico functional analyses of identified rubber tree PR-10 proteins

**Figure 6:**
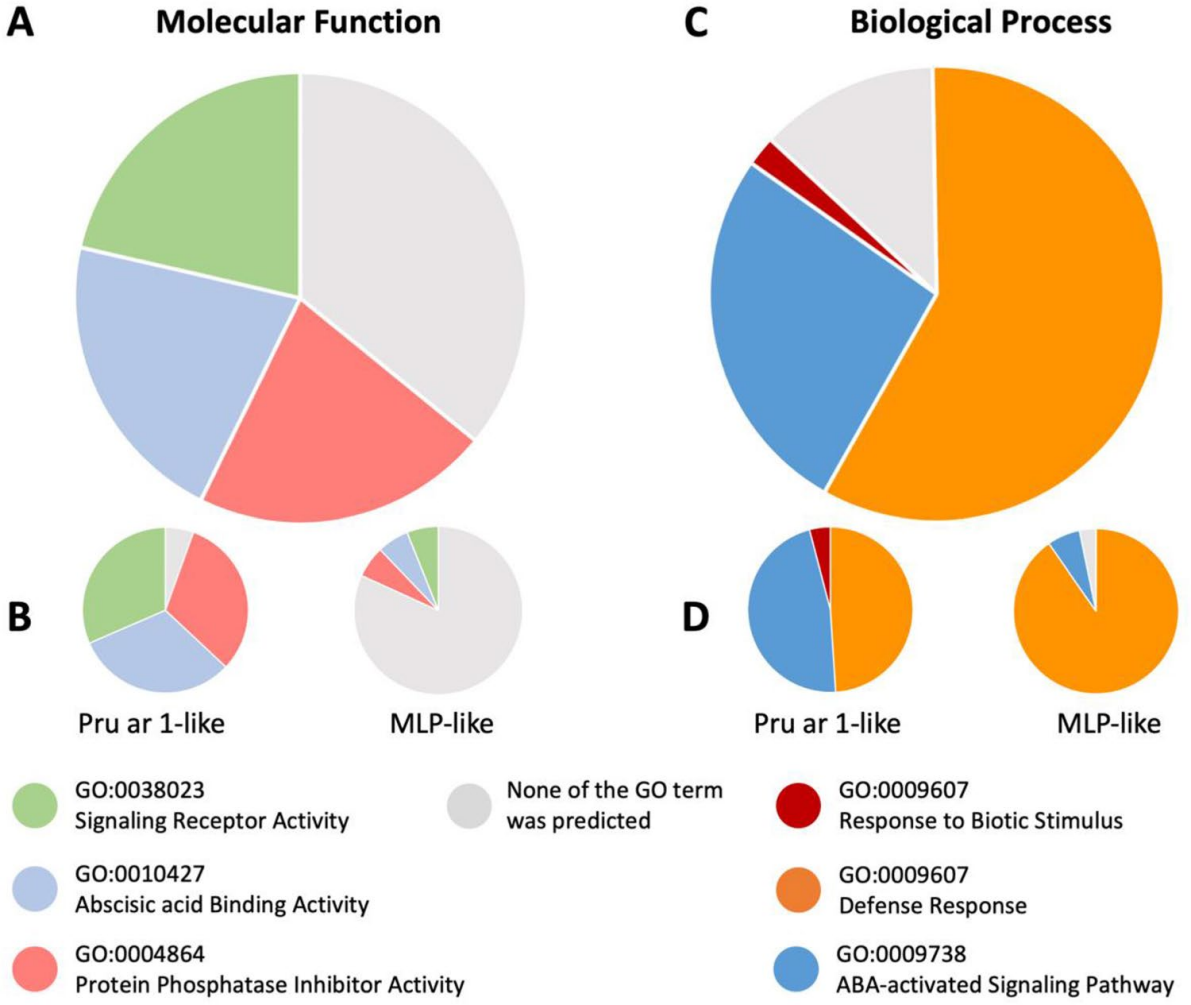
The predicted molecular functions and biological processes of PR-10 proteins identified in rubber tree clone GT-1 according to the GO terms in the UniProt database. The molecular functions (**A**) and the biological processes (**C**) of 67 identified PR-10 proteins from rubber tree clone GT-1 were analyzed. The molecular functions (**B**) and biological processes (**D**) of each of the two main protein subgroups (the Pru ar 1-like subgroup and the MLP-like subgroup) were also compared.

A total of 67 PR-10 proteins from rubber tree cultivar GT-1 with UniProt accessions (Table 1) were acquired for the reported gene ontology (GO) terms in the database (Table S9). Considering all 67 proteins as a representative set of rubber tree PR-10 proteins, the molecular functions and biological process were found to vary by protein (Fig. 6). Three main molecular functions, namely, protein phosphatase inhibitor activity (GO:0004864), abscisic acid binding activity (GO:0010427), and signaling receptor activity (GO:0038023), were found to be associated with rubber tree PR-10 proteins (Fig. 6A), especially in the Pru ar 1-like subgroup (Fig. 6B). However, many of the members of the MLP-like protein subgroup (Fig. 6B) and all of the members of the uncharacterized protein subgroup were found to have no reported molecular function. In terms of biological processes, most of the rubber tree PR-10 proteins showed a role in defense response (GO:0006952), as well as in the ABA-activated signaling pathway (GO:0009738) (Fig. 6C). Most of the members in the MLP-like subgroup showed only a role in defense response, while members of the Pru ar 1-like protein subgroup showed roles in both defense response and the ABA-activated signaling pathway (Fig. 6D). Having a role in responding to biotic stimuli (GO:0009607) was reported in some Pru ar 1-like proteins. Members of the uncharacterized protein subgroup have no reported biological processes.

**Figure 7:**
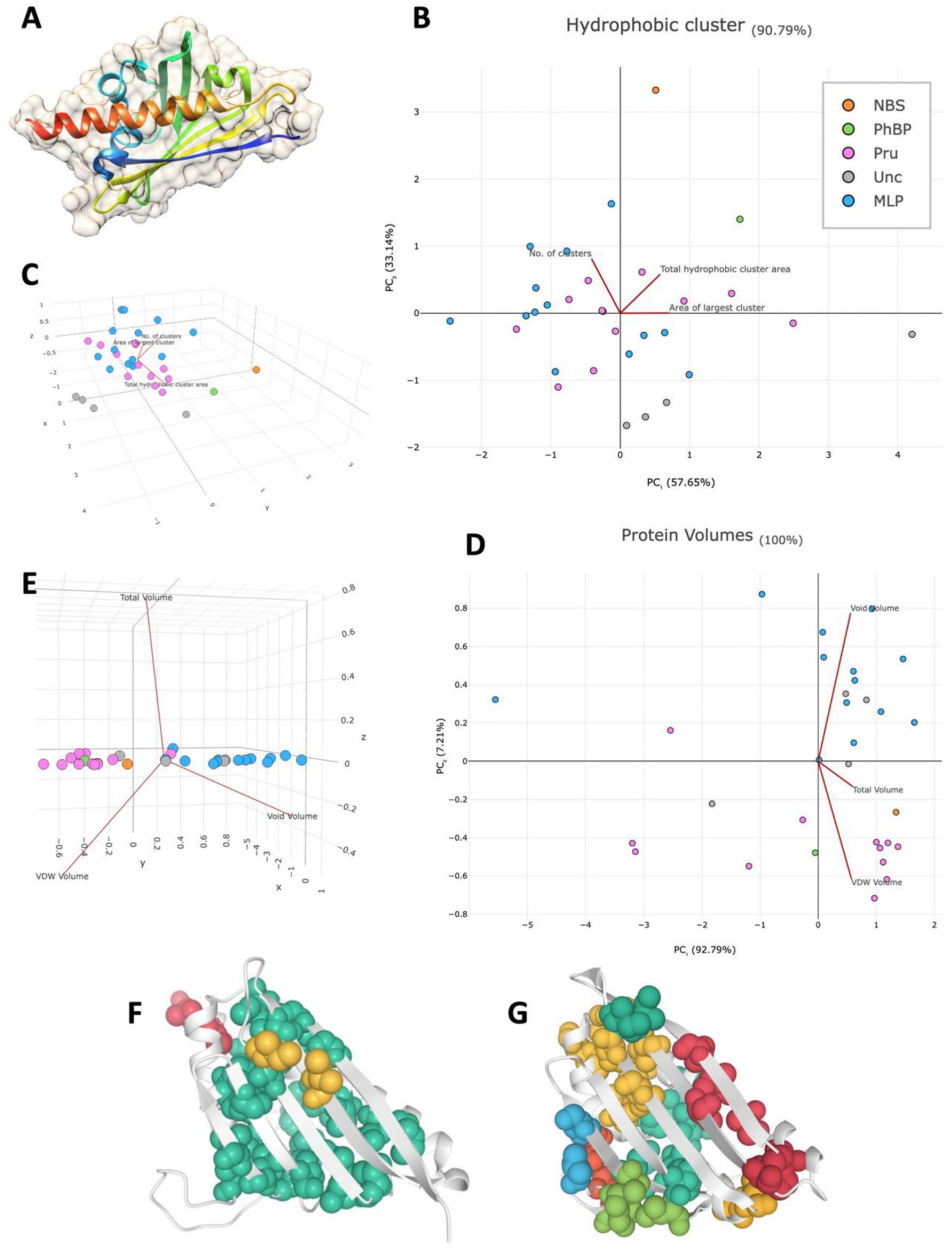
Analyses of volumes and hydrophobic clusters of AlphaFold2-predicted rubber tree PR-10 protein structures with typical Bet v 1 folding. A typical Bet v onefold is found in rubber tree PR-10 proteins. The Bet v 1 topology is highlighted as a dark blue β at the N-terminal followed by the blue α_1_-β_6_ and the red C-terminal α-helix (**A**). The light tan area showed the predicted protein surface. The AF-predicted structure was downloaded from UniProt accession A0A6A6MW69 and visualized by UCSF Chimera. Principal Component Analysis (PCA) plots showing the hydrophobic clusters (**B**, **C**) and protein volumes (**D**, **E**) of rubber tree PR-10 protein structures. The plots were generated using the predicted hydrophobic clusters via ProteinTools and predicted volumes via ProteinVolume for a total of 31 protein structures, including 13 MLP-like proteins, 12 Pru ar 1-like proteins, 4 uncharacterized proteins, a norbelladine synthase protein, and a phytohormone-binding protein (Table S11). The detailed PCA analysis is in Table S12. Diagrams showing the predicted protein structure with predicted hydrophobic clusters of the uncharacterized protein with the largest hydrophobic cluster area (mint color) (UniProt accession A0A6A6NJK4) (**F**) and the norbelladine synthase protein with the highest number of hydrophobic clusters (9 clusters) (UniProt accession A0A6A6M0B6) (**G**).

The AlpaFold2-predicted 3D structure of 67 PR-10 proteins was available for each of the UniProt accessions. Based on manual observation (Table S10), we found that 31 of them possess the typical Bet v 1 folding pattern of the PR-10 family (Fig. 7A), whose topology consists of a β-α_1_-β_6_ structure and a C-terminal α-helix^2^. Some non-typical Bet v 1 folds were found in all subgroups, such as double Bet v 1 folds (as found in the MLP-like protein accession KAF2316804.1 and the Pru ar 1-like protein accessions KAF2289131.1, KAF2308301.1), Bet v 1 folds with an extended C-terminal (as found in the MLP-like accessions KAF2316787.1, KAF2316790.1), those with extended C- and N-terminals (as found in the uncharacterized protein accession KAF2309849.1), or misfolded versions (as found in the MLP-like protein accession KAF2305531.1) (Table S10). To compare the structural characteristics among predicted structures of PR-10 protein subgroups without bias from the extensive extension of amino acid residues, the 31 proteins exhibiting the typical Bet v onefold were analyzed for protein volume and hydrophobic clusters.

Using the ProteinVolume server, the void volume, Van der Waals (VDW) volume, and total volume of each predicted protein structure were assessed (Table S11). The total volumes of rubber tree PR-10 protein subgroups ranged from 16,837 Å^3^ to 214,842 Å^3^, led up to 92.79% variance in PCA analysis (Fig. 7D, Table S12). When comparing the two main subgroups, MLP-like proteins and Pru ar 1-like proteins, these protein subgroups showed differ void volume (PC_2_ in Fig. 7D) but not total volume (PC_1_ in Fig. 7D) and van der waals volume (z-axis in Fig. 7E). When calculating the proportion of void volume relative to total volume, void volume was found to account for more of the total protein volume in the MLP-like subgroup compared to the other subgroups.

The internal cavity of the predicted rubber tree PR-10 protein structures was analyzed for hydrophobic cluster number and area using the ProteinTools server. Overall, the proteins tended to exhibit one of two patterns: a lower number of clusters with larger cluster areas (Fig. 7F) or a higher number of clusters with smaller cluster areas (Fig. 7G). The two main subgroups, MLP-like proteins and Pru ar 1-like proteins, showed no clear difference in their hydrophobic cluster characteristics. Interestingly, four uncharacterized proteins showed the lowest numbers of clusters and the largest cluster areas of up to 3527 Å^2^ (Figs. 7B, 7F). Moreover, a phytohormone-binding protein, a norbelladine synthase protein, and uncharacterized proteins showed distinct hydrophobic cluster characteristics compared to those two major subgroups.

## Discussion

### Members of the PR-10 protein family in rubber tree genomes were identified

In this study, homologous proteins related to the PR-10 protein family were identified and confirmed using several steps (Fig. 1), including query sequence acquisition of PR-10 proteins of model plant species from the Pfam database (PF00407), a BLASTp search against the NCBI database, an HMMscan search, and protein accession matching in the UniProt database. PR-10 protein members were identified from two rubber tree genomes available in the NCBI database, namely, cultivar GT1 and isolate MT/VB/25A 57/8 (Table S1–S8). The most recent genome assembly of isolate MT/VB/25A 57/8^30^ is now being used as a reference genome for the rubber tree *H. brasiliensis* in the NCBI database instead of the former one from clone Reyan7-33–97^42^, which used Illumina sequencing data alone to gather the fragmented genome assemblies. A genome assembly from one of the best rubber tree cultivars, cultivar GT1, previously provided an informative rubber tree genome^29^. They used Illumina sequencing coupled with PacBio assembly to obtain the genome and then anchored the genome assembly to 18 pseudochromosomes via single-molecule real-time sequencing (SMRT) and Hi-C technology. Most of the annotated rubber tree PR-10 proteins in this study were acidic proteins with low molecular weight, and the lack of signal peptides on the protein sequences (Tables S6, S7) confirmed that PR-10 members are small cytoplasmic proteins in plant cells^43^.

Characterization of the identified rubber tree PR-10 genes at the chromosomal level may provide further information for a genetic-based understanding of these proteins and their utilization for the improvement of agronomic traits, especially stress tolerance. We consequently mapped the chromosomal locations of 132 identified PR-10 protein-encoding genes from rubber tree, including cultivar GT1 and isolate MT/VB/25A 57/8 (Fig. 4). The results revealed the distribution of *PR-10* genes on 13 and 11 chromosomes of cultivar GT1 and isolate MT/VB/25A 57/8, respectively. Despite the relatively low gene density of Chr 15 compared to other chromosomes^29^, most of identified *PR-10* genes were shown to cluster there, accounting for almost half of the identified PR-10 proteins in the genomes (Fig. 4). Previously, the conserved gene synteny among rubber tree chromosomes was analyzed and showed paralogous gene sharing between Chr 8 and Chr 9, and between Chr 15 and Chr 18^29^. These are the four chromosomes enriched with PR-10 protein-encoding genes observed in our study, especially cultivar GT-1. These chromosomes may be crucial for PR-10 genes and hence are the perfect candidates for improving rubber tree traits related to PR-10 functional phenotypes.

### The identified rubber tree PR-10 proteins showed a potential role in plant defense responses

A number of previous reports have collectively proven the important roles of PR-10 proteins in diverse aspects of plant functioning. The PR-10/Bet v 1 protein family consists of small intracellular pathogenesis-related proteins involved in several cellular functions in plants and their responses to environmental changes, especially in defense against phytopathogens. Despite the small size of typical PR-10 proteins, they have been shown to bind small molecules in their hydrophobic cavity formed by the Bet v 1  fold (Fig. 7A). According to a review by Aglas and colleagues^3^, three main groups of molecules (flavonoids, cytokinins, and sterols) are linked to probable roles of PR-10 proteins in plant metabolite biosynthesis, host defense, as well as growth and development. PR-10 proteins from various plant species are also well-known for their enzymatic activity in cleaving RNA. Several reports have proven such ribonuclease activity and linked it to PR10-mediated defense in plants^43–47^. Although there was no ribonuclease activity observed in the zucchini MLP-PG1 protein, the protein still showed an enhanced host defense in *MLP-PG1*-overexpressed plants^48^. As not all PR-10 proteins have ribonuclease activity, it is believed that their role is not always linked to the protein’s contribution to host defense^43^.

In addition to ribonuclease, PR-10 proteins have been suggested to exhibit at least 8 other distinct enzymatic activities^4^. For example, PR-10 proteins have been shown to exhibit activity similar to neopinone isomerase in opium poppy^49^ and to beta-1,3-glucanase in *Ma*PR-10^50^.

PR-10 proteins have been tested for defensive roles via overexpression in the model plants *Arabidopsis thaliana* and tobacco. For instance, the moss PpPR-10 protein increased immunity in Arabidopsis against *Pythium irregulare*^51^. The corn *Zm*PR10.1 protein expressed in Arabidopsis reduced necrosis and chlorosis by *Pseudomonas syringae* DC3000^52^. In rice, genes encoding PR-10 proteins (*OsPR10a/PBZ1, OsPR10b, JIOsPR10,* and *RSOsPR10* genes) are known to upregulate in response to infection by *Magnaporthe grisea* pathogenic fungi^53–55^, suggesting a defensive role of PR-10 protein in rice. Moreover, the *Os*Bet v 1 protein from rice was one of the defense protein targets suppressed by the root-knot nematode-derived effector *Mg*MO237^56^. The *Os*Bet v 1 protein was recently proven to confer resistance to rice against *Meloidogyne graminicola* nematode infection through the activity of peroxidase enzymes^57^. In resistant grapevine, a cluster of genes in the Bet v 1/PR-10 protein family was highlighted as a defense-responsive candidate^58^.

**Figure 8:**
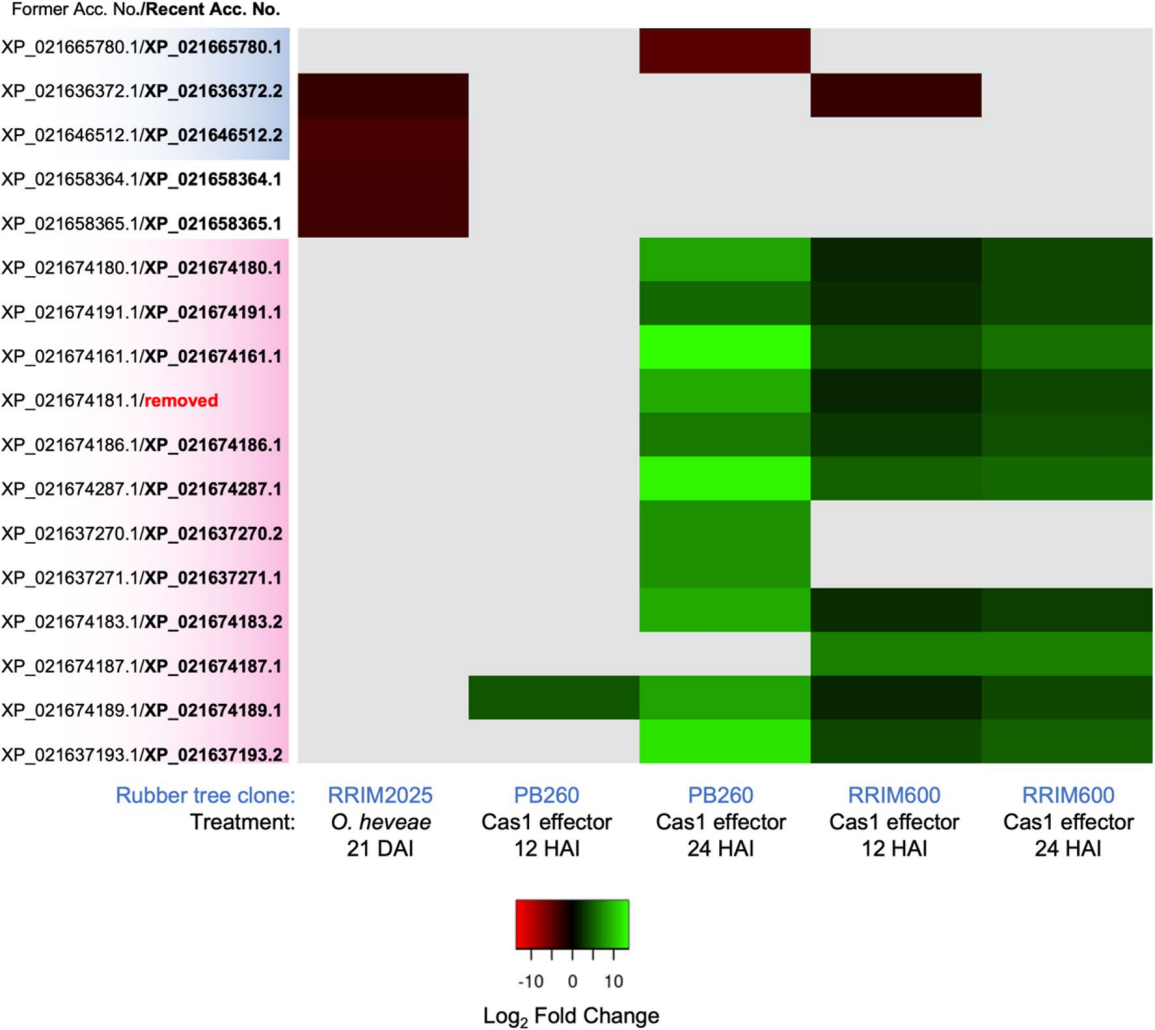
A heatmap showing the transcriptomic profile of some identified rubber tree PR-10 proteins in response to *Oidium heveae*^25^ and to the Cas1 effector from *Corynespora cassiicola*^19^. The identified PR-10 protein accessions in this study were manually searched against the two transcriptome datasets, which were acquired from the available supplementary results of these articles. Note that the protein accessions from these datasets were annotated to the former reference genome of clone Reyan7-33–97^42^, hence, the accessions were updated here according to the recent reference genome of isolate MT/VB/25A 57/8^30^. Then the Log_2_ fold change of the matched candidate gene accessions was used for generating the heatmap by using Heatmapper (http://www.heatmapper.ca/). Missing data is shown in gray. The accession numbers of each protein subgroup are color-coded according to the phylogenetic tree in Figure S1.

In rubber trees, members of the PR-10 protein family were identified in this study and their associated GO terms indicate potential roles in plant defense, response to biotic stimuli, and response to the defense-related ABA phytohormone (Fig. 6C). Some previously identified PR-10 proteins exhibiting a response to biotic stress were recently reported (Fig. 8). In response to the necrotrophic cassicolin Cas1 fungal effector from the pathogenic fungi *Corynespora cassiicola*, the 12 Pru ar 1-like major allergen proteins of rubber tree were significantly upregulated in both rubber tree clones PB260 and RRIM600, which were higher at 24 h after inoculation (HAI) than 12 HAI^19^ (Fig. 8). Contrary to the Pru ar 1-like protein, two MLP-like proteins from another major subgroup that we identified in this study were downregulated in response to the fungal effector treatment. Also, the MLP-like proteins, as well as the two uncharacterized proteins (former names LOC110648447 isoform X1 and X2, then recently renamed as LOC131168728 and LOC110648447) were downregulated proteins identified in powdery mildew (*Oidium heveae*)-infected rubber tree leaves^25^ (Fig. 8). These findings highlight the likely role of rubber tree PR-10 proteins in plant defense, which is a conserved role of the protein family across the plant kingdom.

### Classification of rubber tree PR-10 proteins into subgroups

By using MEME tool analysis for the protein motifs, 10 novel motifs were predicted to be enriched in the set of 132 identified rubber tree PR-10 proteins examined in this study (Supplementary 1). All 10 newly discovered motifs were subjected to motif comparison against the PROSITE database via the Tomtom tool in order to investigate if any of them are well-known protein motifs with previously reported functions. However, the results of established motifs that were significant matches with each of the 10 novel motifs seem inconclusive (data not shown), as no perfectly identical motifs were found. Such findings are not uncommon, as novel motifs are predicted based only on the set of protein sequences provided and using the specific criteria, we set for the MEME tool. Together with the phylogenetic analysis performed in this study, the location of the predicted motifs on each PR-10 protein sequence using the MEME tool clearly reflected the common characteristics of protein members in each subgroup (Figs. 2, 3, and S2).

Based on their reported names from rubber tree isolate MT/VB/25A 57/8, the evolutionary relationships established by ML, and the novel motifs identified by MEME tool analysis (Figure S2), the 132 identified PR-10 proteins from two rubber tree cultivars were classified into two main subgroups, which were the major allergen Pru ar 1-like protein subgroup and the major latex (MLP)-like protein subgroup, and three other minor subgroups. Although all the identified proteins from rubber tree cultivar GT1 and some proteins from isolate MT/VB/25A 57/8 did not have annotated names, the results of the phylogenetic analysis and motif analysis in this study support the similarities of the protein subgroups between the two cultivars. Therefore, the hypothetical proteins in the PR-10 protein family from cultivar GT1 and isolate MT/VB/25A 57/8 have now been annotated into the proper protein subgroups based on the current analysis (Tables S6 and S7).

The evolutionary relationships of the rubber tree PR-10 proteins found in this study resemble the recent distance-based relationships reported by Morris and colleagues^4^, which were adapted from the classic evolutionary tree of plant PR-10 proteins by Radauer and colleagues^2^. The PR-10/Bet v 1-like families include the plant PR-10 group (dicot PR-10 s, monocot PR-10 s, and conifer PR-10 s), the phytohormone-binding and norcoclaurine synthase (NCS) protein group, the moss PR-10 and MLP protein group, and the polyketide cyclase-like outgroup^4^. More resemblance was noticed in the relationships among grape PR-10 proteins, in which Zhang and colleagues classified grape PR-10 proteins into five groups: major allergen Pru av 1 protein/STH-2 proteins, MLP-like proteins, ABA receptors, S-norcoclaurine synthase (NCS)-like proteins, and uncharacterized proteins^26^. The dominance of the subgroups Pru ar 1-like proteins and MLP-like proteins found in this study among the identified PR-10 homologs in the rubber tree could reflect their putative importance in the species. However, the enzyme-like PR-10 protein found in the recent rubber tree genome is norbelladine synthase (NBS) but not NCS-like protein, which was identified in grape.

Additional analyses of the GO terms and structural features (i.e., volumes and hydrophobic clusters) of the identified PR-10 proteins in rubber tree were performed to see if they could reflect any differences among protein subgroups in addition to the sequence similarity-based phylogenetic tree (Fig. 7). Although these analyses were limited in that they were performed with only some of the identified PR-10 proteins, and came only from rubber tree cultivar GT-1, their available protein data and AlphaFold2-predicted structures using UniProt demonstrate that they are representative of rubber tree PR-10 proteins, as the proteins from cultivar GT-1 were found across all five subgroups of rubber tree PR-10 proteins (Figure S1). Differences in structural features were found, especially in the bulkier and larger hydrophobic clusters in the cavity of an uncharacterized protein (accession KAF2325348.1) (Fig. 7G).

The following discussion will focus on the characteristics of each subgroup, together with the previously reported roles of the subgroup members.

#### Pru ar 1-like major allergen protein subgroup

Allergen proteins derived from plants could play a role in plant defense, serving as a mechanism to protect themselves from pathogens and herbivores. Mal d 1, the major allergen from apple (*Malus domestica*), exhibits an immunological relationship with Bet v 1, the major birch pollen allergen^59,60^. Additionally, Mal d1 functions as a defensive protein, belonging to the group 10 of pathogenesis-related proteins. Plants express it in response to various stressors, including pathogen infection, chemical exposure, wounding, and stressful environmental conditions^61^. Proteome analysis of pathogen-responsive proteins in apple leaves revealed that Mal d 1 was induced by *Alternaria alternata*^62^.

A total of 15 allergen classes found in rubber trees have been reported, including some defense-related proteins like hevamine (Hev b 14) and serine protease inhibitor (Hev b 15)^63^. While the Pru ar 1 allergen has not been reported as a latex allergen in rubber trees, this allergen group has been specifically associated with the defense mechanisms of rubber trees^19^.

Major allergen Pru ar 1-like proteins are homologs of the fruit allergen Pru ar 1 proteins, first identified in apricots (*Prunus armeniaca*)^64^. These proteins were recently found to show an association with necrosis that resulted from hybrid incompatibility in ornamental Japanese flowering cherries^65^. In *Citrus grandis*, Pru ar 1-like proteins increased in leaves in response to copper-induced treatment^66^. Interestingly, the Pru ar 1-like gene of arabica coffee (LOC113766162) was suggested as a crucial genomic locus in coffee leaf rust resistance and coffee yield^67^. The proteins in this subgroup were found to be highly upregulated in two rubber tree clones against the fungal effector Cas1 (Fig. 8), in which the expression of a Pru ar 1-like protein (accession XP_021674161.1) was up to 13.58 fold in rubber tree clone PB260. These findings suggest the putatively important role of Pru ar 1-like proteins in plant stress, especially in terms of biotic response.

Based on the motif comparison results by Tomtom analysis, none of the 10 novel motifs predicted by the MEME tool were matched with the signature pathogenesis-related protein Bet v 1 (PATHOGENESIS_BETVI) motif (ID: PS00451) in the PROSITE database (Figs. 5B, 5C). We then further manually searched for this motif in each of the 132 identified rubber tree PR-10 protein sequences against the PROSITE database. Interestingly, only 14 proteins in this study showed the consensus sequence of the PATHOGENESIS_BETVI motif (Fig. 5A), and all of them are Pru ar 1-like proteins. The consensus sequence of the PATHOGENESIS_BETVI motif seems mostly conserved among the related proteins in the subclade, but the overlooked proteins have amino acid substitutions or insertions/deletions (InDels) in the motif sequence (Fig. 5A).

Taken together, the previously reported transcriptomic data suggest a crucial role for the Pru ar 1-like protein subgroup in rubber tree defense. The presence of recognized defense-related protein motifs in the members of this subgroup only, out of the 5 subgroups revealed in this study, highlights Pru ar 1-like proteins as front-runner candidates for further improving rubber tree tolerance to several diseases.

#### Major latex (MLP)-like protein subgroup

Another subgroup of identified rubber tree PR-10 proteins are the MLP-like proteins, which are designated by different names, including MLP28-like, MLP31-like, MLP328-like, MLP329-like, MLP34-like, and MLP423-like proteins (Figure S2; Table S6). Dicot plant species contain more MLP homologs than monocot plants, especially Solanaceous plants, which are reported to have up to 56 *MLP-like* genes in potato (*Solanum tuberosum*) and 27 *MLP-like* genes in tomato (*S. lycopersicum*)^26^. In this study, we identified 70 MLP-like proteins in the rubber tree genomes (Figs. 3, S2).

MLP proteins have been recognized as ripening-related (RRP) proteins in fruits^2,68^ and there have been several reports of their roles in tolerating plant stress and defense against plant pathogens. MLP proteins were found in the phloem juice of melon after infection by the cucumber mosaic virus^69^. The mulberry *Mu*MLP329 protein was found to mediate the JA-signaling pathway in defense against phytoplasma^70^. In Arabidopsis, MLP28 was increased in response to Alternaria infection^71^. Additionally, two genes in the rubber tree that were differentially expressed upon exposure to the ethylene-stimulated tapping dryness (TPD) disease condition were homologous to the two loci of the MLP423 gene in Arabidopsis^72^.

The resistance of *N. benthamiana* to *Potato virus Y* (*PVY*) was reported to be improved by *Nb*MLP28 overexpression either transiently or constitutively^73^. The sugar beet proteins *Bv*MLP1- and *Bv*MLP2- expressed in Arabidopsis led to reduced infection by *Rhizoctonia solani*^74^. Overexpression of the cotton *Gh*MLP28 in tobacco lowered disease incidence by *Verticillium dahliae*^75^, and the zucchini MLP-PG1 expressed in tobacco reduced the lesion areas of *Botrytis cinerea* infection^48^.

In a systemic review by Morris and colleagues^4^, the lack of identified plant-derived ligands for the previously characterized MLP proteins was mentioned. The predicted biological process of MLP-like proteins in this study showed members with a term of defense response (GO:0006952), differed to the Pru ar 1-like subgroup whose biological process also has an ABA-activated signaling pathway (GO:0009738) (Fig. 6D). Therefore, ABA-related molecules could be hypothesized to be the ligand that binds to the large hydrophobic cavity of Pru ar 1-like proteins, but the ligands related to the relatively-larger-void-volume MLP-like proteins may be the others.

Unlike the Pru ar 1-like subgroup, none of the members in the MLP-like protein subgroup contained the signature motif of the Bet v 1/PR-10 protein family (Fig. 5). Hence, the defense-related functions of MLP-like proteins may depend on other unidentified motifs and related to other mode of action in plant cells. Previously, the total flavonoid content was doubled in Arabidopsis plants overexpressing the cotton MLP gene^76^, suggesting the positive contribution of MLP proteins in plant metabolite synthesis. Moreover, the activity of MLP-like proteins was also recently suggested by Lichman and colleagues^77^. The comparative genomics of catnip (*Nepeta* spp.) revealed several MLP-like proteins with unknown functions, which accelerate the cis–trans cyclization of the intermediate step in the nepetalactone biosynthesis pathway. However, the detailed molecular mechanisms of MLP-like proteins remain to be discovered, as they preferably focused on the nepetalactone gene cluster^77^. Together with the commonly reported upregulation of MLP-like genes and proteins upon plant defense response, a deeper characterization of the molecular mechanisms of MLP-like proteins will broaden our understanding of this protein subgroup.

#### Norbelladine synthase (NBS) and phytohormone binding (PhBP) protein subgroup

In this study, only one NBS protein was identified in the reference rubber tree genome, isolate MT/VB/25A 57/8, and another hypothetical protein accession, KAF2306724.1 from cultivar GT-1, also shared the evolutionary cluster on the ML tree as well as the motif characteristics with this NBS protein (Figs. 2, S2). The former reference genome of the rubber tree from cultivar Reyan7-33–97 was annotated another enzyme-like PR-10, S-norcoclaurine synthase (S-NCS)-like protein (NCBI accession XP_021656580.1), in the rubber tree genome. The rubber tree S-NCS-like protein was identical to the hypothetical protein accession KAF2306724.1, which is clustering as an NBS homolog in the present analysis. However, the accession of the S-NCS-like protein (XP_021656580.1) was now suppressed according to the updated reference genome re-annotation in the NCBI database^30^.

The evolutionary connection between the NBS and PhBP proteins appears to mirror that of the well-established Norcoclausine synthase (NCS) protein within the PR-10 protein family seen in various plants^2,4^. The protein analogous to NBS closely aligns with PhBP proteins in a distinct minor subgroup located next to the primary two subgroups of the rubber tree PR-10 protein (Figs. 2, 3, S2). Nonetheless, each protein is believed to serve distinct functions: enzymatic functions are more common in NBS and likely S-NCS-like proteins, whereas PhBP proteins predominantly exhibit phytohormone-binding capabilities.

NCS proteins are involved in the biosynthesis of benzylisoquinoline alkaloids in plants^78–80^. In *Thalictrum flavum*, (S)-norcoclaurine was synthesized by the catalytic activity of the NCS protein^81^. According to the pathway BioCyc ID: PWY-3581, the product of the enzymatic reaction is an intermediate in the (S)-reticuline biosynthesis pathway, which is the substrate for the synthesis of several secondary metabolites. Among the six recognized metabolite-biosynthetic activities performed by PR-10 proteins^4^, NCS activity is the very first bona fide enzymatic activity that has been characterized in 7 species from Papaveraceae, 1 species from Berberidaceae, and 3 species from Ranunculaceae^5–8^.

NBS functions as an enzyme, facilitating the transformation of 3,4-dihydroxybenzaldehyde and tyramine into norcraugsodine. This is then converted into norbelladine by the action of the norcraugsodine reductase enzyme. Notably, norbelladine is a crucial metabolite involved in various molecular alterations during the subsequent production of alkaloids, particularly Amaryllidaceae alkaloids like the Alzheimer’s medication, galanthamine^82^. The role of NBS in norbelladine creation was previously confirmed in two specific Amaryllidaceae plants^83,84^.

These findings further confirm the role of the PR-10 protein family as a contributor to plant metabolite biosynthesis. However, the NCS-like and NBS proteins found in rubber tree is likely to be a minor functional gene that shares the same noncatalytic ancestor with the above-mentioned catalytic NCS proteins from three plant families^85,86^. The emergence of a PR-10 protein with NCS activity was phylogenetically located in Ranunculales species prior to the diversification of the families Papaveraceae and Ranunculaceae^27^. In rubber trees (family Euphorbiaceae, order Malpighiales), therefore, the NCS-like protein identified in this study may lack NCS activity similar to the NCS-like proteins from *Arabidopsis thaliana*, *Oryza sativa*, *Betula pendula*, and *Pinus monticola*^87,88^. Additionally, the presence of NBS activity in rubber trees and other plant families beyond the Amaryllidaceae might require verification.

In addition to enzymatic activities, PR-10 proteins may participate in metabolite biosynthesis by regulating the flux of intermediates using protein binding activity ^4^. The structural analysis of several previously reported PR-10 proteins has revealed the potential of the large hydrophobic cavity in binding small molecules, such as quercetin-3-O-glucuronide, myricetin, and ( +)-catechin, which bind strawberry Fra a proteins^89^, and quercetin-3-O-sophoroside (Q3OS), which binds Bet v 1 proteins^90^. Previous articles have suggested the importance of the entrance to the cavity in ligand specificity, including the major entrance framed by the long C-terminal α helix and loops L3, L5, and L7, and the minor entrance framed by the β-sheet 1 and α helix 3^4,91,92^. Here, the investigation of the AlphaFold2-predicted structure of the two rubber tree proteins in this subgroup (NCS-like KAF2306724.1 and PhBP KAF2285953.1) showed a considerably broad major entrance to the internal cavity. Future experiments investigating the phytohormone binding affinity of these rubber tree PhBP proteins will provide a clearer role for this PR-10 protein subgroup in rubber trees, such as PhBP proteins from other species that have been shown to bind natural and synthetic cytokinins or gibberellins^93,94^.

#### Uncharacterized protein subgroup

Several uncharacterized proteins were identified as members of the rubber tree PR-10 protein family in this study based on the criteria used (Fig. 1). However, further investigation of their characteristics should provide more information on their putative roles as well as their proper classification into the main subgroups. The MLP-like subgroup and the uncharacterized proteins shared a conserved N-terminal motif 5 and central motif 2. Interestingly, the phylogenetic analysis coupled with the novel motif prediction clearly suggested that the uncharacterized proteins are clustered together as a minor subgroup with an altered motif characteristic from the closely related MLP-like protein subgroup (Figs. 3, S2).

The hydrophobic cluster in one uncharacterized protein showed an exceptionally large area compared to the other identified rubber tree PR-10 proteins in this study (Fig. 7F), suggesting the potential binding ability of hydrophobic molecules. Although functional annotation via GO terms was lacking for this subgroup of PR-10 proteins, we recently noticed the sharp upregulation of a novel uncharacterized PR-10 protein (LOC110648447; former accessions XP_021658364.1, XP_021658365.1, and XP_021658366.1) in rubber tree leaves after being challenged by the white root rot fungus *Rigidoporus microporus*^20^. The biological process of LOC110648447 was thus predicted to be crucial for defense response, response to biotic stimuli, and ABA-activated signaling pathways, which is similar to the predicted biological process of rubber tree PR-10 proteins in general (Fig. 6C). In response to powdery mildew, this uncharacterized protein (LOC110648447) showed reduced expression in leaves, which are the infected tissue^25^ (Fig. 8). Several previous reports have suggested ABA as a positive regulator of rubber tree defense against pathogens^95,96^, but ABA biosynthesis in rubber trees can be suppressed by the EqCSEP01276 effector from the powdery mildew pathogen^97^. From the difference in expression patterns between the systemic response to root disease and the local response to foliar disease, we hypothesize that ABA may be a key phytohormone in the expression of the uncharacterized protein LOC110648447.

## Conclusions

To summarize, the PR-10 protein family members in the rubber tree *H. brasiliensis* have been systemically identified from the genomes available in the NCBI database for the first time. Their identification and classification based on protein characteristics, including motif analysis and evolutionary relationships, revealed two major groups, Pru ar 1-like major allergens and major latex-like (MLP) proteins. These have been previously acknowledged for their significance in plant defense. A chromosomal mapping was undertaken, indicating potential chromosomal positions for PR-10 genes. The identification and in silico characterization of rubber tree PR-10 proteins here paves the way for advanced research on these proteins in this important crop species. Moreover, deepening our grasp on defense-related proteins may offer alternative strategies to develop rubber tree clones with desirable traits in the future.

### Supplementary Information

Supplementary Information 1.

Supplementary Information 2.

Supplementary Information 3.

## Data Availability

The data for each step from the in silico identification generated during this study are included in this published article via supplementary Tables S1–S12. The raw data of the selected transcriptomic profiles can be accessed via the supplementary tables of cited articles.
