# Supplementary material for: Genome-wide identification of rubber tree pathogenesis-related 10 (PR-10) proteins with biological relevance to plant defense
Source: Sci Rep. 2024 Jan 11;14:1072. doi: 10.1038/s41598-024-51312-3 (PMC10784482; doi:10.1038/s41598-024-51312-3)
Supplement: Supplementary file 2 — Supplementary Information 2. [file 41598_2024_51312_MOESM2_ESM.docx]

**Supplementary 1**

**1) The 10 discovered motifs by MEME tool**

| Motif | Motif consensus sequence | E-value of motif prediction | Sites | width |
| --- | --- | --- | --- | --- |
| 1 | IPKILPQAIKSVEVLEGDG | 1.4e-938 | 135 | 19 |
| 2 | IEAIDKENKTITYSVIEGDPLKDYKE | 6.6e-916 | 115 | 26 |
| 3 | PKGQGSLAKLTLEYEKLNEBVPVPHKYLDF | 1.5e-837 | 53 | 30 |
| 4 | KISYEIKFVASPDGGSICKTTSKYYTKGBF | 1.2e-762 | 47 | 30 |
| 5 | EVZJKSGPEKFLGLFKAQE | 9.3e-578 | 118 | 19 |
| 6 | GPGSIKKWTFTEGG | 7.1e-534 | 97 | 14 |
| 7 | MGVVTYELEVTSPVPPAKLFKALVLDSDIL | 9.2e-278 | 20 | 30 |
| 8 | AYLLANPDAY | 2.5e-253 | 47 | 10 |
| 9 | DEEKKIVTJIGLEGDVFKIYKVYTVIWELT | 8.8e-209 | 15 | 30 |
| 10 | VISVTKDIDAGJSK | 4.3e-145 | 40 | 14 |

| Motif | Motif consensus sequence | Motif logo |
| --- | --- | --- |
| 1 | IPKILPQAIKSVEVLEGDG | 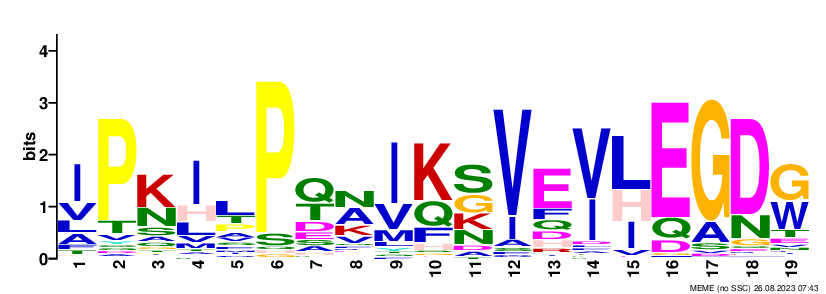 |
| 2 | IEAIDKENKTITYSVIEGDPLKDYKE | 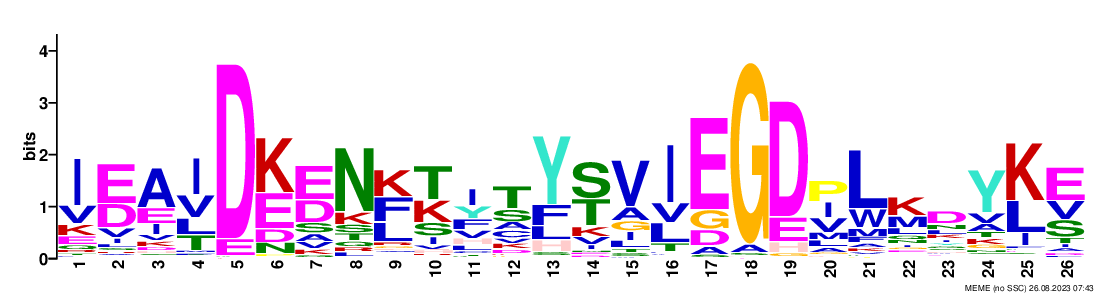 |
| 3 | PKGQGSLAKLTLEYEKLNEBVPVPHKYLDF | 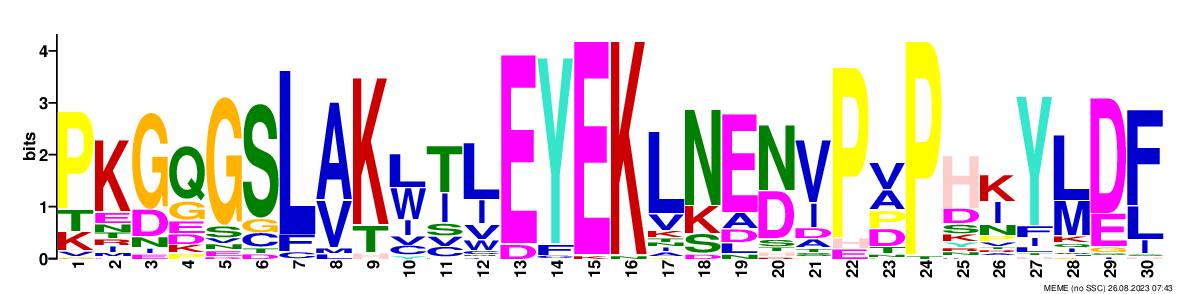 |
| 4 | KISYEIKFVASPDGGSICKTTSKYYTKGBF | 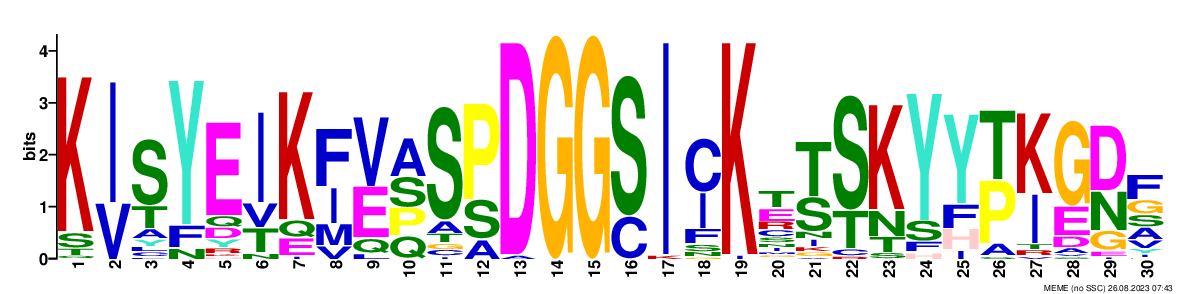 |
| 5 | EVZJKSGPEKFLGLFKAQE | 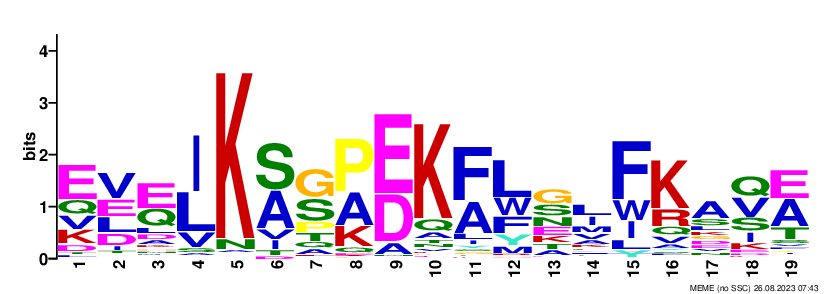 |
| 6 | GPGSIKKWTFTEGG | 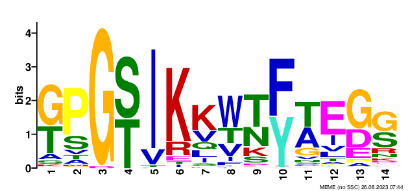 |
| 7 | MGVVTYELEVTSPVPPAKLFKALVLDSDIL | 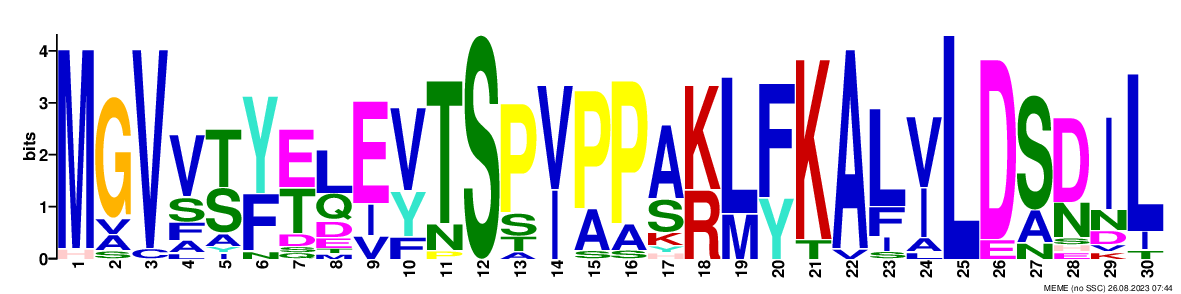 |
| 8 | AYLLANPDAY | 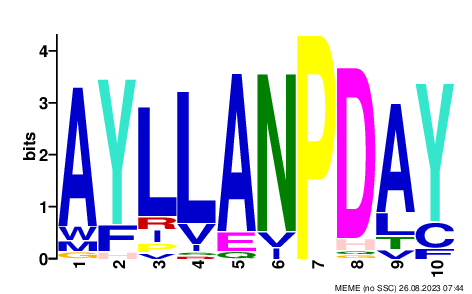 |
| 9 | DEEKKIVTJIGLEGDVFKIYKVYTVIWELT | 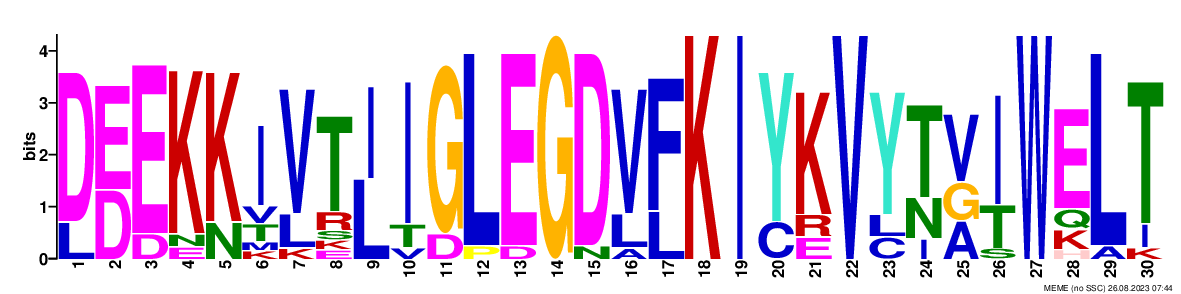 |
| 10 | VISVTKDIDAGJSK | 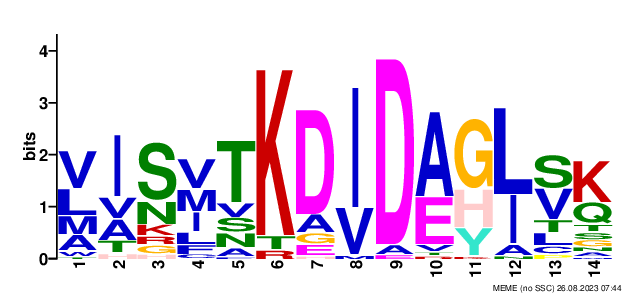 |

**2) The motif locations**
