# Supplementary material for: Genome-wide identification of rubber tree pathogenesis-related 10 (PR-10) proteins with biological relevance to plant defense
Source: Sci Rep. 2024 Jan 11;14:1072. doi: 10.1038/s41598-024-51312-3 (PMC10784482; doi:10.1038/s41598-024-51312-3)
Supplement: Supplementary file 3 — Supplementary Information 3. [file 41598_2024_51312_MOESM3_ESM.docx]

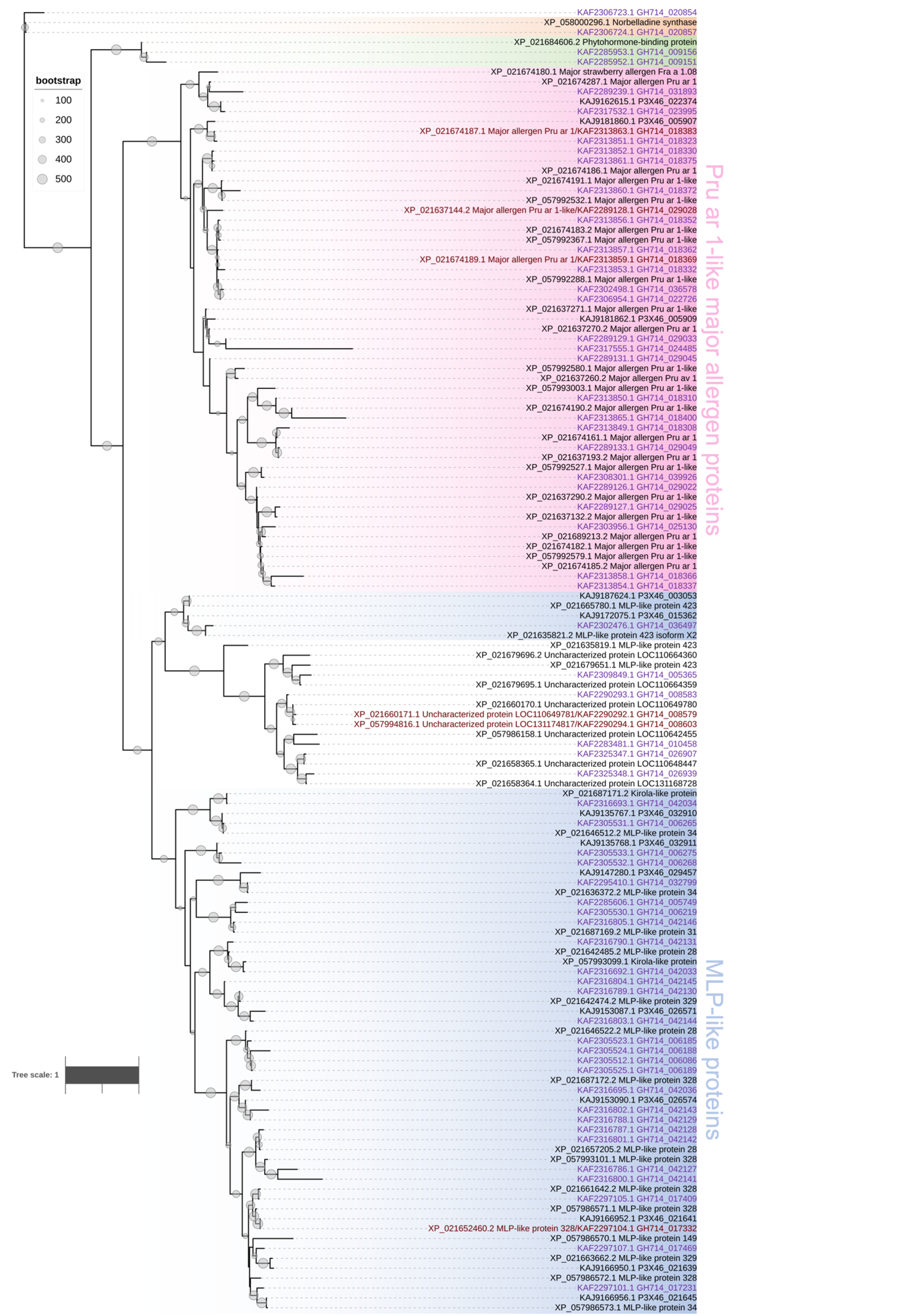


Figure S1: The evolutionary relationship of identified PR-10 proteins from two rubber tree cultivars, GT-1 and MT/VB/25A 57/8, inferred using maximum likelihood (ML) with 500 bootstraps. The protein accessions from cultivar GT-1, MT/VB/25A 57/8, or both cultivars are text-coloring as purple, black, and brown, respectively. Five protein subgroups were identified based on the annotated names: Norbelladine synthase proteins (orange), phytohormone-binding proteins (green), Pru ar 1-like allergens (pink), uncharacterized proteins, and major latex protein (MLP)-like proteins.


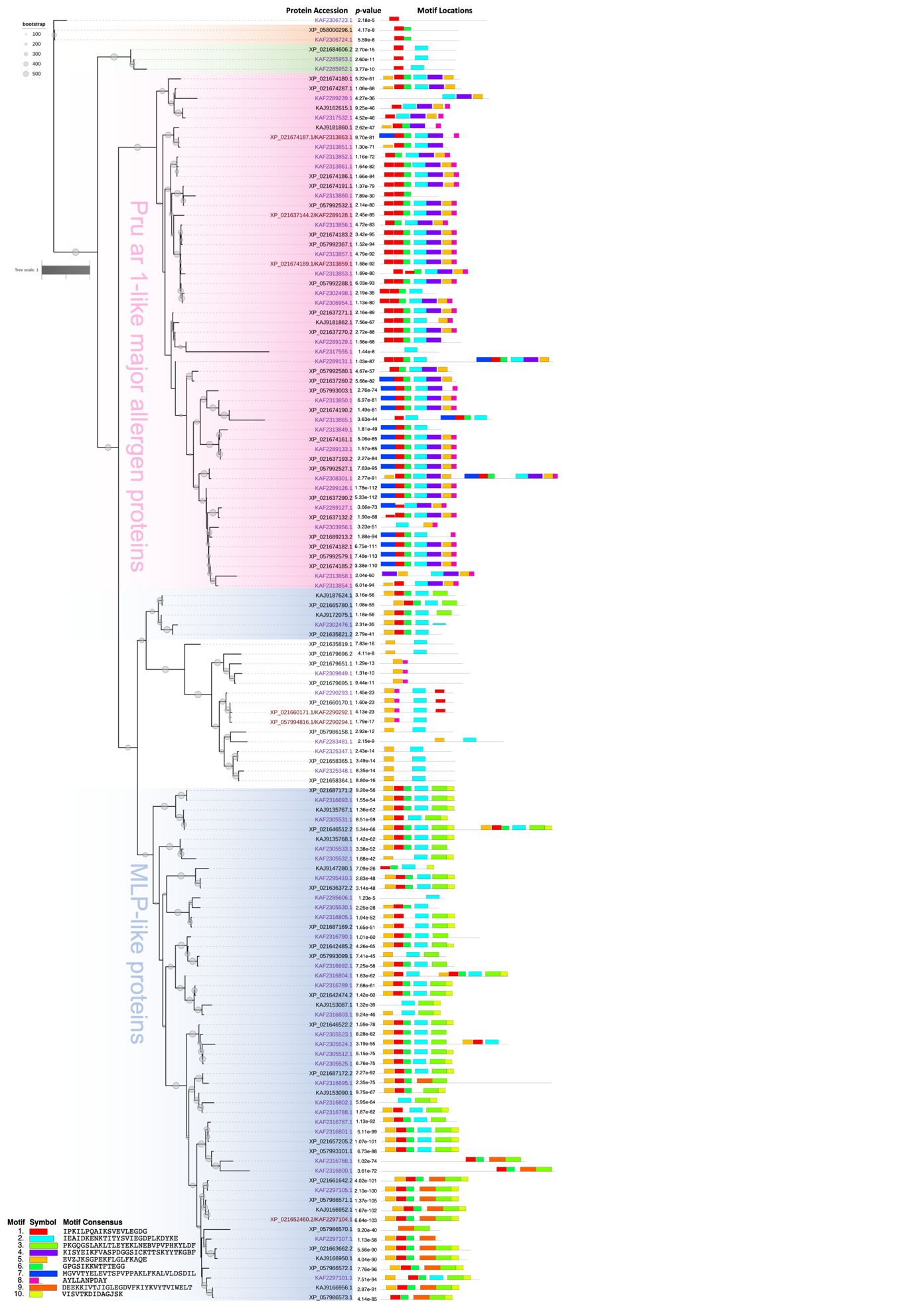


Figure S2: The evolutionary relationship of identified PR-10 proteins from two rubber tree cultivars, GT-1 and MT/VB/25A 57/8, inferred using maximum likelihood (ML) with 500 bootstraps (Figure S1) with the motif analysis by the MEME tool. The 10 novel motifs (bottom left corner), and the *p*-values of motif predictions and the motif locations on the protein sequences are shown to the right of each identified PR-10 protein. A detailed motif analysis by the MEME tool is presented in Supplementary 1.
